# lmp2gro: A Python Tool for Converting LAMMPS Data Files into GROMACS Topologies

**DOI:** 10.1021/acs.jcim.6c01430

**Published:** 2026-07-20

**Authors:** Alexandre Moni Pereira, Jarede da Silva Martins, Marcelo Albuquerque, Luciano T. Costa

**Affiliations:** MolMod-CS, Institute of Chemistry, 28110 Fluminense Federal University, Campus Valonguinho, Centro, Niterói-RJ CEP 24020-141, Brazil

## Abstract

lmp2gro is a Python utility designed to streamline the generation of GROMACS topologies for systems where traditional parametrization is often challenging. Although developed primarily for periodic systems, such as metal–organic frameworks (MOFs), the tool is equally applicable to molecular systems containing a single residue type. The program parses LAMMPS data files, maps force field terms onto the corresponding GROMACS functional forms, and automatically generates all necessary input files. This workflow enables seamless access to all of the capabilities of the GROMACS simulation engine. The versatility of the tool is demonstrated through two case studies: (i) H_2_ adsorption in MOF-5 (IRMOF-1) using the universal force field (UFF) and (ii) the behavior of a water droplet on a silica Q4 surface using the INTERFACE-FF. A step-by-step guide is provided for both applications. The lmp2gro source code and tutorial materials are freely available at https://github.com/AlexandreMoniPereira/lmp2gro.

## Graphical abstract

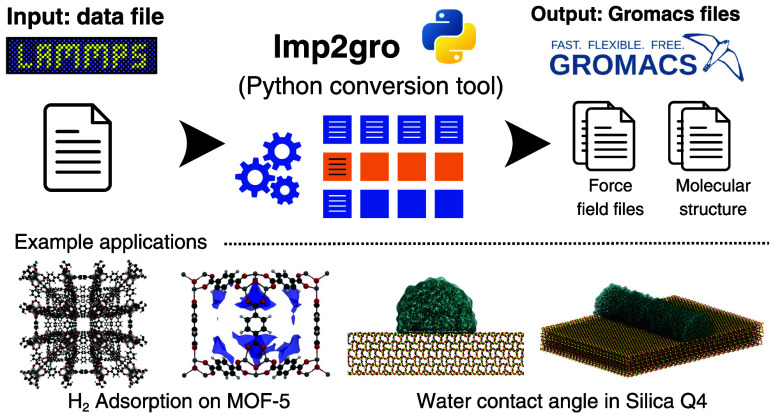

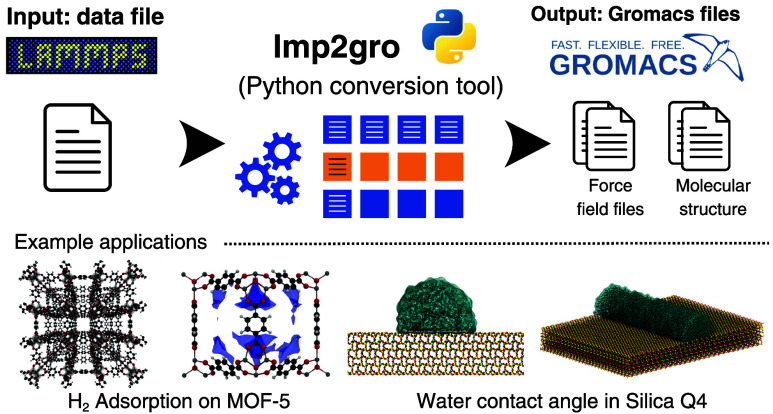

## Introduction

Several critical applications in chemistry and materials science, including catalysis,^1–3^ gas sequestration,^4–6^ and the development of energetic materials,^7–10^ rely on the modeling of extended systems such as polymers, metal–organic frameworks (MOFs), and crystalline solids. To bridge the gap between atomistic-level interactions and macroscopic phenomena, researchers typically employ periodic boundary conditions (PBC). This approach enables the simulation of a representative unit cell to approximate bulk material properties, effectively mitigating the computational costs associated with simulating nonperiodic, large-scale systems.

Although robust workflows exist for the automated generation of small-molecule topologies (through tools such as DLPGEN,^11^ LigParGen,^12^ CHARMM-GUI,^13^ the ATB topology builder^14^ and acpype)^15^, the treatment of periodic systems remains a significant bottleneck. The inherent complexity of defining bonds, angles, and dihedrals across periodic boundaries means that fewer software packages provide general, automated solutions for these architectures, often requiring laborious manual tasks or system-specific scripting.

In response to these challenges, the Large-scale Atomic/Molecular Massively Parallel Simulator (LAMMPS)^16^ has established a prominent role in materials science, creating a versatile ecosystem of tools designed for periodic topology construction. This includes built-in utilities such as msi2lmp, amber2lmp, and ch2lmp, which convert existing force field formats into LAMMPS data files. Furthermore, several specialized software packages have been developed to generate LAMMPS-ready structures directly, including the OVITO visualizer,^17^ lammps_interface,^18^ cif2lammps,^19^ and the expanded capabilities of CHARMM-GUI.^13^

In contrast, the GROMACS simulation suite^20^ offers fewer native options for the automated generation of periodic topologies. To date, OBGMX^21^ is a notable utility in this domain, providing a pathway to generate universal force field (UFF)^22^ topologies directly for GROMACS-compatible periodic architectures, such as MOFs. GROMACS is widely recognized as one of the most computationally efficient molecular dynamics engines.^20^ Although its development has historically prioritized biomolecular systems—such as proteins, lipids, and nucleic acids—its optimized algorithms for computing nonbonded interactions and its comprehensive suite of analysis tools have significantly broadened its appeal. Furthermore, the extensive ecosystem of compatible software, including Grace and MDAnalysis,^23^ combined with its well-documented and standardized file formats, makes GROMACS a compelling choice for researchers in diverse fields, seeking to leverage high-performance simulation and postprocessing capabilities.

To take advantage of the complementary strengths of these simulation platforms, several interoperability tools have been developed. In addition to the previously mentioned msi2lmp, amber2lmp, and ch2lmp, independent utilities such as GRO2LAM^24^ facilitate the transition of molecular systems into the LAMMPS environment. Likewise, the all2lmp module as implemented in LUNAR, a LAMMPS pre- and postprocessing toolkit,^25^ assigns bonded parameters and charges to the system, as well as defines the simulation cell and delivers the topology files for LAMMPS, is inspired on msi2lmp. Another example of a more comprehensive effort toward software interoperability is represented by InterMol,^26^ which enables conversion between GROMACS, LAMMPS, DESMOND,^27^ CHARMM,^28^ and AMBER.^29^ However, despite these advancements, a significant procedural gap remains: many of these tools struggle with the specific complexities of periodic connectivity in nonbiological systems. Although we are aware of LAMMPS’ beneficial capabilities for end users, to our knowledge, there is currently no specialized tool designed that robustly automates the conversion of LAMMPS data files for extended periodic architectures—such as crystalline solids—into functional, GROMACS-compatible topologies. Furthermore, existing solutions fail to support the OpenMM package^30^ while simultaneously accounting for parameters along the periodic boundary conditions.

To address this limitation, we have developed lmp2gro, a Python-based utility that serves as a bridge between these two widely used engines. Our workflow utilizes the flexible building capabilities of LAMMPS to generate, replicate, and organize the initial periodic topology. Subsequently, lmp2gro translates this information into GROMACS-compatible formats, enabling researchers to perform high-performance MD simulations while maintaining full access to the extensive GROMACS analysis and visualization ecosystem.

## Software Design and Methods

lmp2gro is a Python code that generates single-molecule topologies for the GROMACS simulation package. It was primarily designed to handle periodic structures with bonds, angles, and dihedrals that are present through periodic boundary conditions. Usually, setting up the topology for such systems and with this particular issue is left entirely to the user that, in general, is not an expert to deal with this detail. In addition, only a few independently developed tools are currently available.

lmp2gro requires detailed preprocessing of the structures in the form of a fully prepared LAMMPS data file. This is not always a trivial task; therefore, we provide not only data files compatible with lmp2gro, but also tutorials for the case studies that assist the user in the creation of the initial data files.

The usage of lmp2gro is straightforward and consists of the following command:

python3 lmp2gro.py data.lammps_data_file [more flags]

Upon execution, lmp2gro follows the workflow depicted in Figure 1. First, the code reads the LAMMPS data file, parsing all coefficient and structural directives. The data-collector.py module is responsible for processing the LAMMPS data and generating data frames for pandas^31^ that contain relevant information. It also calls fit-equations.py when it is necessary to convert the parameters into the appropriate functional forms and units used in GROMACS.

**Figure 1:**
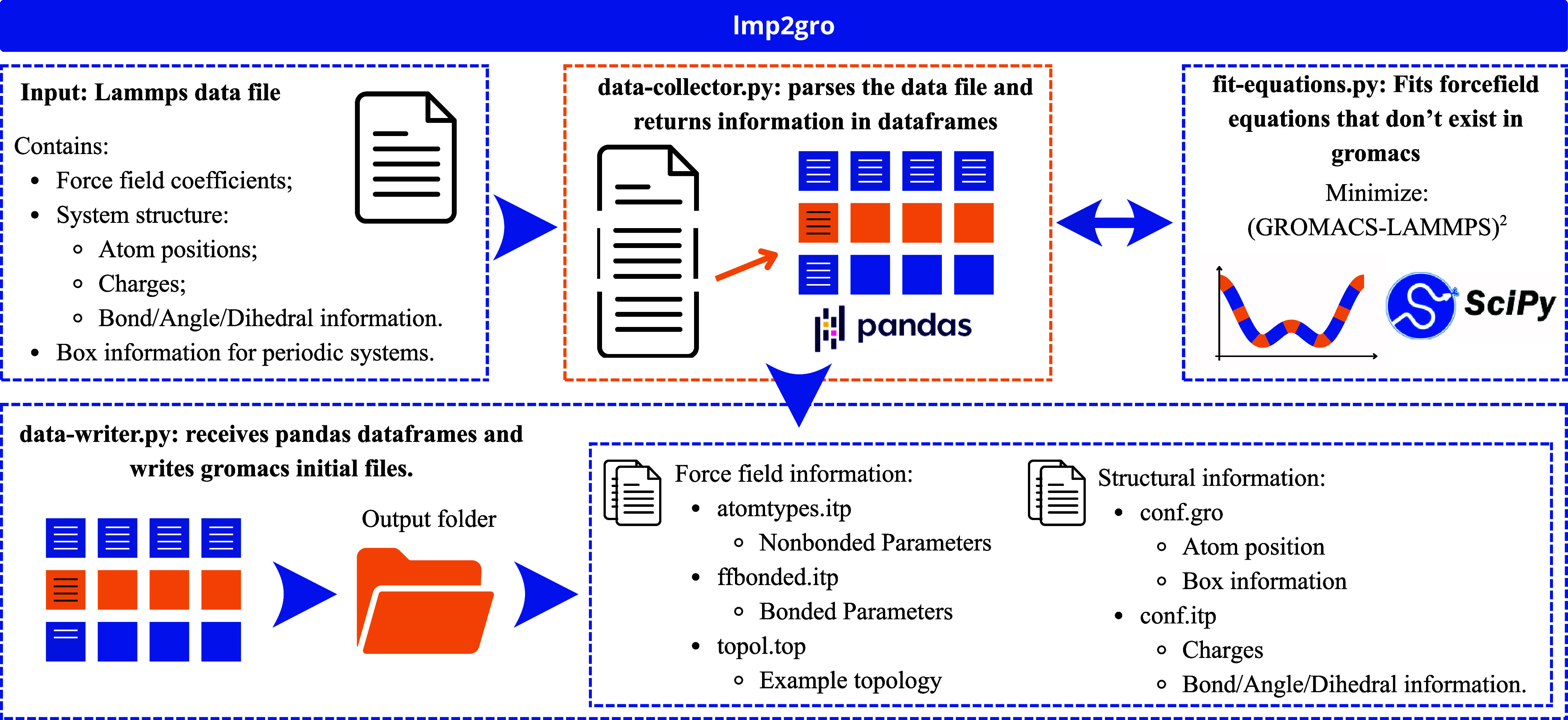
Workflow of the lmp2gro tool, illustrating the steps required to convert LAMMPS data files into GROMACS-compatible inputs.

The fitting routines use SciPy^32^ minimize with the default method (usually BFGS)^33–36^ to minimize the Least Squares Method between original and fitted functions, thus obtaining the best representation of the functional forms of LAMMPS in terms of GROMACS potentials. Additional functional forms can be easily incorporated into this module. NumPy^37^ is also used throughout the previous steps to handle array transformations. Once the data are structured and organized, lmp2gro calls the data-writer.py module, which generates the output directory that contains all the system information required to run GROMACS.

lmp2gro assumes that the units are defined in the “real” LAMMPS style. Accordingly, during the conversion process, distances are transformed from angstroms to nanometers, and energies are transformed from kcal/mol to kJ/mol to ensure compatibility with GROMACS. Before submitting a data file, it is essential to verify that the units are defined in the “real” style, as the use of alternative unit systems may lead to inaccuracies in the output.

One of the first tasks of the script is to parse the LAMMPS header, identifying the numbers of atoms, bonds, angles, dihedrals, and impropers. If any of the corresponding entries are not present, they are set to zero. The data-collector then processes each directive (e.g., “Bonds”) using the corresponding count to iterate over the entries and retrieve the relevant information. This approach is also applied to the type numbering associated with the coefficient directives.

Currently, lmp2gro supports a selected set of interaction potentials from LAMMPS data files (listed below). Because each of these supported functional forms requires a unique number of numerical constants, the tool automatically detects the potential type based on its parameter count.

1. **Bonds**: LAMMPS harmonic style is converted to the GROMACS harmonic potential.
2. **Angles**: The following LAMMPS styles are supported: cosine-periodic, Fourier, and harmonic.
  Cosine-periodic and harmonic styles are converted to the GROMACS harmonic form; however, this significantly limits the representation of the cosine-periodic potential, and its use is therefore not recommended.
  The Fourier style is converted to the GROMOS96 functional form in GROMACS, providing an accurate fit.
3. **Dihedrals**: The following LAMMPS styles are supported: harmonic and OPLS.
  The harmonic style is converted to the GROMACS periodic function;
  The OPLS style is converted to the GROMACS Fourier function.
4. **Impropers**: The following LAMMPS styles are supported: harmonic, Fourier, and cvff_harmonic.
  Harmonic and Fourier styles are converted to the GROMACS harmonic improper form; The cvff style is converted using the GROMACS Fourier function.

The Supporting Information presents the functional forms employed in both LAMMPS and GROMACS, together with graphical comparisons of the corresponding potential energy functions and tables containing the converted parameters. These results demonstrate the accuracy of the fitting and conversion procedures. Additionally, for the case studies, parity plots are provided for each individual bonded energy contribution, further validating the preservation of the original force-field behavior after conversion.

The original UFF formulation^22^ employs a cosine-periodic angle potential; however, the original work also describes how this form can be converted to a Fourier representation. A simple modification to the *cif2lammps* tool can force the use of the Fourier style; detailed instructions for this procedure are provided in GitHub repository.

GROMACS restricts improper torsion interactions to a specific harmonic functional form. Although these energies can be approximated using periodic dihedral potentials, this mapping merges improper energy contributions into the broader dihedral energy term. Consequently, this prevents the isolated decomposition and reporting of improper torsion energies within the simulation output, representing a trade-off between functional flexibility and energy decomposition. In this work, we adopt the improper harmonic form to fit the functions used in UFF, despite the slightly reduced fitting accuracy. This choice allows the user to retain the ability to analyze improper energy contributions independently.

With respect to the nonbonded parameters, atomic charges are directly transferred, as they are already expressed in compatible units. The Lennard-Jones potential is assumed to follow the form 12–6 ϵ−σ and is converted accordingly to ensure compatibility with the GROMACS format.

### Optional Arguments

The following optional arguments provide additional flexibility when generating GROMACS input files, allowing the user to customize residue naming, output organization, and the treatment of bonded interactions. These options are particularly useful when adapting LAMMPS-generated data files to different force fields or simulation workflows.

#### Residue Name

By default, the residue name is set to “UNL” (unknown ligand). A custom name can be specified using the --resname or -r flag:

python3 lmp2gro.py data.lammps_data_file -r RES

#### Output Directory

The output is saved in a folder named after the input file. To define a custom directory, use the --folder flag:

python3 lmp2gro.py data.lammps_data_file -r RES --folder folder_name

In some force fields, such as Clay-FF,^38^ all interactions, or the majority of them, are treated as nonbonded. Depending on the method used to generate the data files, bonded terms may still be present. For this reason, we develop the --clean argument. When used alone, it identifies and removes bonded parameters for which all values are zero. Additionally, specific bonded interactions can be removed by explicitly providing their indices, as shown below:

python3 lmp2gro.py data.lammps_data_file -r RES --folder folder_name \ --clean -b “1 2” -a “1 2 3” -d “1” -i “1 2”

The flags -b, -a, -d, and -i correspond to the indices of bonds, angles, dihedrals, and impropers to be removed, respectively. In the next sections, we will present practical applications to highlight the usefulness of lmp2gro tool.

## Case Study 1: H_2_ Adsorption in MOF-5

One of the most important highly porous and widely investigated metal–organic frameworks is IRMOF-1 or MOF5.^39–42^ Their structure file was obtained from the CCDC database under entry number 938394 ^43^ as deposited by Lock et al.^44^ To obtain an ab initio optimized structure and atomic charges, density functional theory (DFT) calculations were performed under periodic boundary conditions using the Quantum ESPRESSO package (version 6.8),^45^ using default convergence parameters. We used the generalized gradient approximation (GGA) with the Perdew–Burke–Ernzerhof (PBE)^46^ for the exchange-correlation functional. Core electrons and their interactions with valence electrons were described using Projector Augmented-Wave (PAW) method.^47^

The Bader method, as implemented by Henkelman et al.,^48^ was used to compute atomic point charges for MOF-5. Data files for the optimized MOF-5 structure and a version containing Bader charges were created using cif2lammps,^19^ which are available together with lmp2gro.

The lmp2gro tool was then applied to generate GROMACS topologies from the available LAMMPS data files. No atomic charges were used, following the original UFF parameters. Figure 2b shows the final MOF-5 structure composed of 2 × 2 × 2 unit cells derived from the original CCDC CIF file.

**Figure 2:**
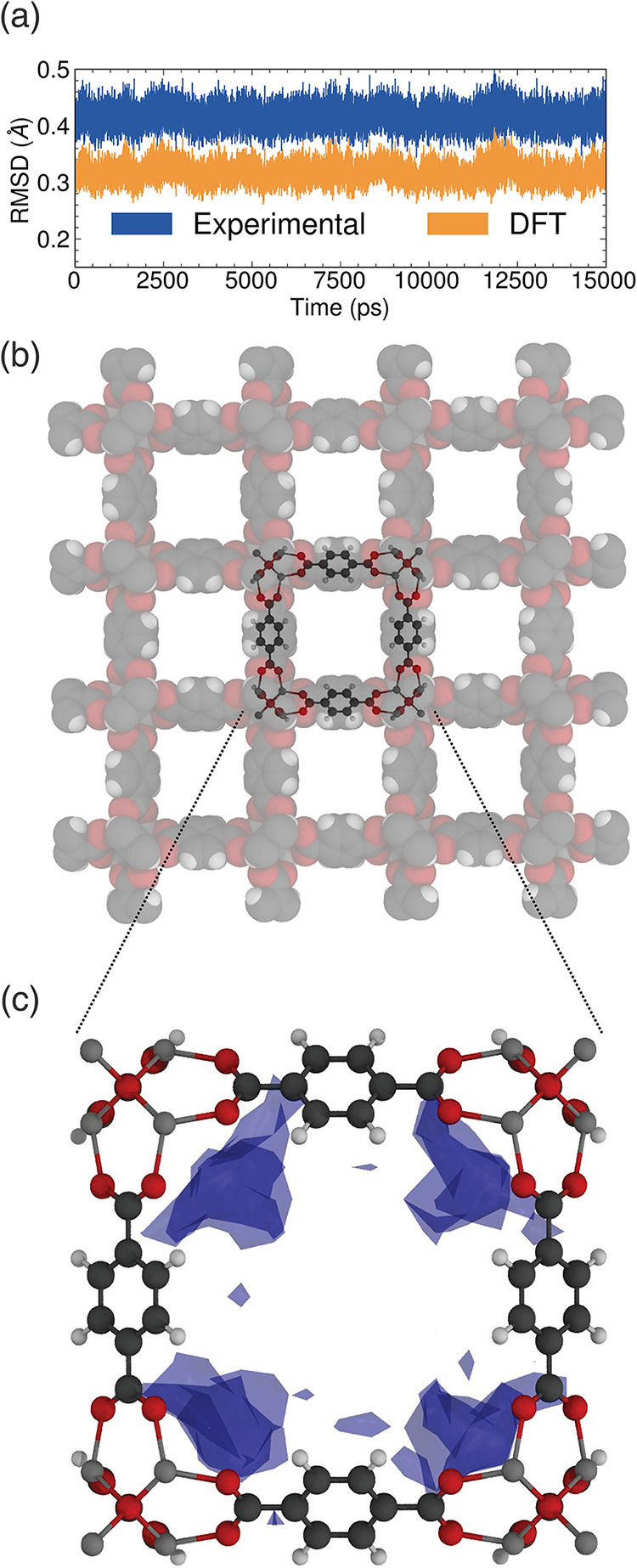
(a) Time evolution of the RMSD of MOF-5 relative to the experimental CCDC structure and the DFT-optimized structure.^43^ (b) Final 2 × 2 × 2 MOF-5 supercell used in the simulations. (c) Density isosurface (blue region) indicating the most probable adsorption regions for H_2_ within the framework.

We performed molecular dynamics (MD) simulations using the GROMACS 2023.3 package.^20^ Energy minimization was carried out using the steepest descent algorithm, followed by a 20 ns NPT simulation performed at 298.15 K. Temperature and pressure were controlled using the V-rescale thermostat^49^ and the C-rescale barostat,^50^ with coupling times of 0.1 and 1 ps, respectively.

The last 15 ns of the structural evolution of MOF-5 were analyzed against the experimental structure of the CCDC and the DFT-optimized structure using the GROMACS RMSD tool. Figure 2a shows the time evolution of these RMSDs results. Garberoglio^21,51^ performed a similar analysis in the OBGMX publication and reported comparable results for MOF-5 simulated with GROMACS: “with a typical RMSD width of 0.4 Å, showing no sign of instability.”

However, the UFF results are in closer agreement with the DFT structure, suggesting that the structures generated by this procedure may serve as suitable starting points for subsequent DFT evaluations within a multiscale simulation framework. It is also important to note that the deviation of DFT structural parameters relative to experimental values is less than 1%.

To assess adsorption capacity, a molecular model of H_2_ was constructed using OBGMX,^21,51^ ensuring consistency in the force field parametrization. Ten H_2_ molecules were subsequently introduced into the MOF pores using the GROMACS insert-molecules utility. The resulting system was subjected to a 20 ns production simulation, strictly adhering to the equilibration and production protocols established for the pure MOF framework. We then applied the MDAnalysis^23^ density tool to identify the most probable adsorption regions, which aligned with previously reported adsorption sites.^52^ The resulting density isosurface is shown in Figure 2.

## Case Study 2: Water Droplet on a Silica Q4 Surface

To demonstrate the application of INTERFACE-FF^53^ in complex interfacial systems, this case study focuses on the interaction of a water droplet with a silica surface. The INTERFACE-FF framework is particularly well-suited for this task, as it offers a comprehensive library of silica models with precisely characterized degrees of hydroxylation (Q2, Q3, and Q4) as established by Emami et al.^54^

Among these models, the Q4 surface was selected to evaluate the water–silica contact angle, a critical metric to assess the wettability of the material. This system serves as a rigorous validation point; its hydrophobic nature and well-documented behavior in the literature^54,55^ make it an ideal benchmark for testing the transferability of force field parameters. The silica Q4 structure was obtained from the INTERFACE database,^53^ and msi2lmp was used to generate the corresponding LAMMPS data file. The CVFF-compatible version of the INTERFACE force field (CVFF-IFF) was selected to ensure compatibility with the standard functional forms supported by GROMACS and the lmp2gro conversion framework. Using LAMMPS (version 22 Jul 2025), the system was replicated seven times along the *x* direction, resulting in a simulation box of approximately 23.3 × 3.4 nm. The slab thickness in the *z* direction was kept as in the original model, while the vacuum region was extended to 100 nm. Figure 3a illustrates the procedure of constructing the system.

**Figure 3:**
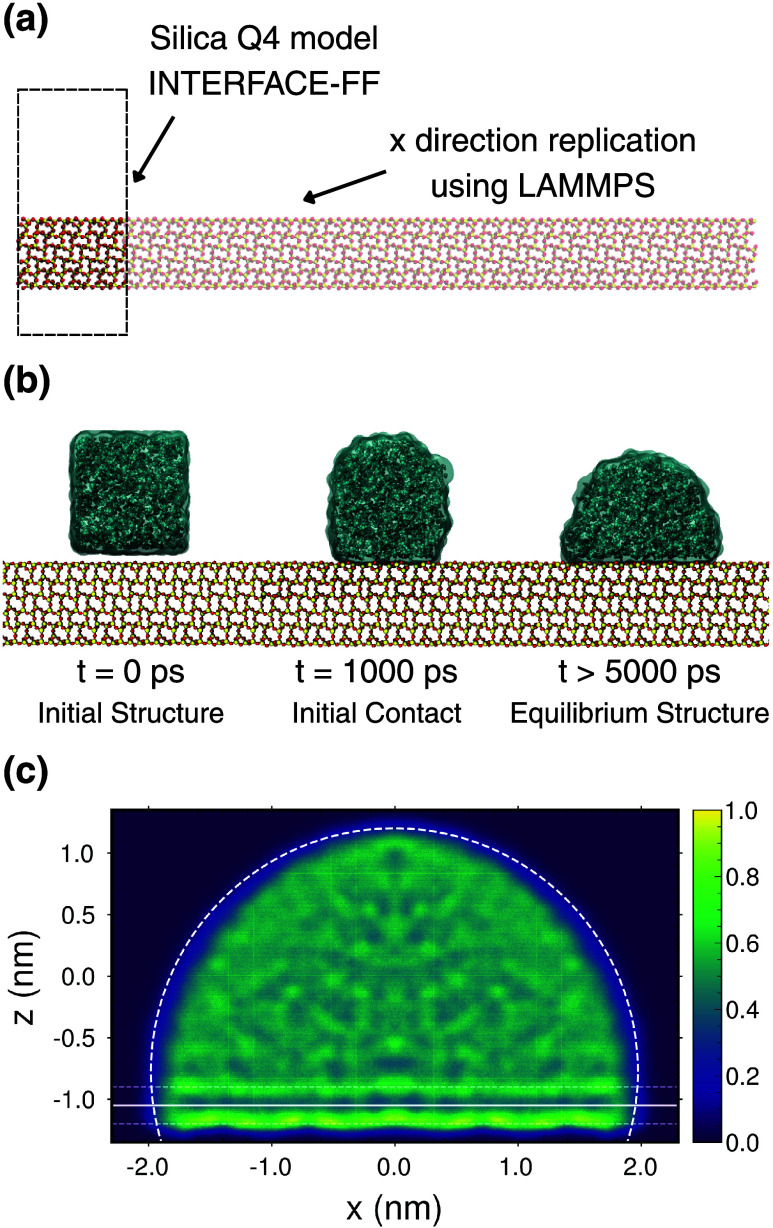
(a) Construction of the silica Q4 surface. (b) Representative snapshots of the system evolution during the molecular dynamics simulation. (c) Density contour map of the water droplet used to determine the contact angle, showing the Gibbs contour, the fitted circle, and the range of baseline positions considered.

A cubic TIP4P/2005 water box of approximately 40 nm^3^ was then generated using Packmol^56^ and placed on the surface, centered along the *x* axis of the simulation box. This setup corresponds to the so-called cylindrical droplet method, which has been widely used in the literature.^55,57^ This approach improves computational efficiency and, as the droplet evolves into a cylindrical shape during the simulation, reducing the influence of line tension effects.

We applied a simulation procedure similar to that described in Case Study 1. The GROMACS 2023.3 package^20^ was used to perform all molecular dynamics simulations. First, an energy minimization was performed using the steepest descent algorithm. Subsequently, a 20 ns NVT simulation was performed at 298.15 K. Temperature was controlled using the V-rescale thermostat,^49^ with a coupling time of 1 ps. Figure 3b shows representative frames of the system evolution.

The last 10 ns of the simulation were used to compute the contact angle. First, MDAnalysis was employed to perform an atomic position count, generating a density profile of the water droplet. The density in the center of the cylinder was taken as the bulk value, and 50% of this value was defined as the Gibbs dividing surface between water and vacuum; this similar definition was used by Liu et al.^55^ Points along the water–vacuum interface were then obtained by identifying positions corresponding to this density. This interface will here be referred to as the “Gibbs contour”.

A circle was then fitted to the Gibbs contour, and the contact angle was calculated relative to a baseline defined at the water–silica interface region. As the definition of this baseline is somewhat arbitrary, a range of 0.3 nm was considered. Figure 3c shows the density contour map, the fitted circle, and the range of baseline positions used in the analysis. The resulting contact angle is 97.95° ± 2.56°, which is in good agreement with the experimental value of 92.1 ± 3.7°.^55^ It is important to note that the uncertainties are obtained using different approaches: in the MD simulations, the error arises from variations in the baseline definition, whereas experimentally it is derived from multiple independent measurements.

## Conclusions

In summary, lmp2gro is an effective tool for converting LAMMPS data files into GROMACS topologies. Its applicability is not inherently limited by the choice of force field, but rather by the availability of compatible GROMACS parametrizations. The code relies primarily on the functional forms available in GROMACS and applies fitting procedures for those that are not directly supported. Additional functional forms can be incorporated into the fit-equations.py module, making the tool extensible.

To demonstrate the applicability of lmp2gro, we developed step-by-step workflows for two representative systems. In the first case, the structural properties of MOF-5 were evaluated and compared with experimental data obtained from X-ray diffraction, as well as with higher-level calculations based on density functional theory (DFT). Furthermore, H_2_ adsorption sites were calculated and found to be in good agreement with the reports of the literature. In the second case, the contact angle between water and a silica surface was calculated, showing good agreement with the experimental values within the associated uncertainties.

## Supplementary Material



## Data Availability

The code and data underlying this study, together with a supplementary lmp2gro manual, are freely available on GitHub at https://github.com/AlexandreMoniPereira/lmp2gro.git. The repository also includes tutorials for case studies 1 and 2, and additional step-by-step case studies are currently under development and will be made available.

## Notes

Mathematical expressions of LAMMPS and GROMACS functional forms, visual comparisons of force field functionals for UFF and INTERFACE-FF (CVFF version), and parity plots for molecular dynamics validation of case studies 1 and 2 (PDF)
