# Supplementary material for: lmp2gro: A Python Tool for Converting LAMMPS Data Files into GROMACS Topologies
Source: J Chem Inf Model. 2026 Jul 20;66(15):8698–704. doi: 10.1021/acs.jcim.6c01430 (PMC13471423; doi:10.1021/acs.jcim.6c01430)
Supplement: Supplementary file 1 [file ci6c01430_si_001.pdf]

Supporting Information: lmp2gro: A Python Tool for Converting LAMMPS Data Files into GROMACS Topologies

Alexandre Moni Pereira,* Jarede da Silva Martins, Marcelo Albuquerque, and Luciano T. Costa

MolMod-CS, Institute of Chemistry, Fluminense Federal University, Campus Valonguinho, Centro, Niterói-RJ, CEP 24020-141, Brazil

E-mail: amoni@id.uff.br

Potential Comparison

For this section, we demonstrate the potential formulations used for both GROMACS and LAMMPS, and display selected fits comparing formulations from Cases 1 and 2. Thus, we focus on both the Universal Force Field[S1](Case 1) and the INTERFACE Force Field[S2] (Case 2). Mathematical functional formulation herein described are available on both LAMMPS and GROMACS manuals.[S3,S4]

To directly compare the bonded parametrization in both simulation packages, we run LAMMPS[S5] simulations using only the solid phases of the case studies (e.g., the bulk structure of MOF-5 for Case Study 1 and the Q^4 silica surface for Case Study 2).

From the obtained LAMMPS trajectories, we used VMD[S6] to convert the coordinates into the GROMACS[S7] `.trr` format. Then, we used the `gmx mdrun -rerun` tool to compute the energies with the exact same structures generated by LAMMPS. Finally, we plot parity plots for each individual bonded energy component to analyze the agreement.

Case 1

Bonds

The LAMMPS harmonic bond potential is defined by:

$$E = K(r - r_0)^2 \tag{1}$$

while in GROMACS, the corresponding functional form is:

$$E = \frac{1}{2}K(r - r_0)^2 \tag{2}$$

Consequently, the core operational difference lies within the definitions of the force constant (K), where the LAMMPS parameter intrinsically incorporates the 1/2 scaling factor ($K_{\mathrm{GROMACS}} = 2 \times K_{\mathrm{LAMMPS}}$ after unit conversion). Furthermore, because the fundamental

unit systems differ between both molecular dynamics engines (energy in kcal mol^{-1} vs. kJ mol^{-1}, and distance in Å vs. nm), explicit parameter conversion is required.

Figure S1 illustrates the potential energy alignment using the converted parameters for the Zn_5 - O_3 bond pair in Case 1.

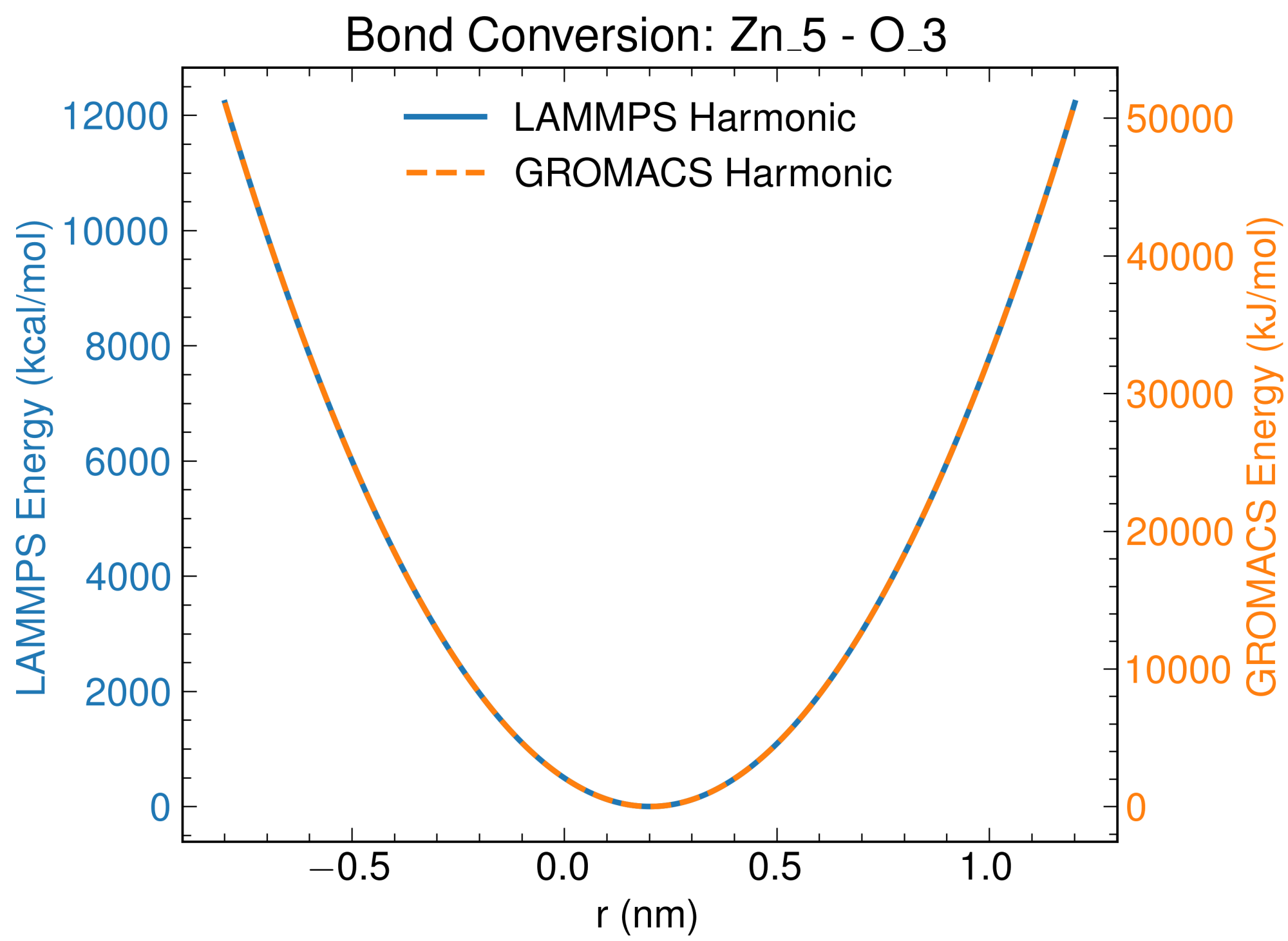

Figure S1: Comparison of the converted harmonic bond potential profiles between LAMMPS and GROMACS for the Zn_5 - O_3 interaction.

The remaining mapped and converted parameters are summarized in Table S1.

Table S1: Mapped equilibrium distances (r_0) and force constants (K) converted between LAMMPS and GROMACS configurations.

		LAMMPS		GROMACS	
**Atom i**	**Atom j**	r_0 **(Å)**	K **(kcal mol^{-1} $Å^{-2}$)**	r_0 **(nm)**	K **(kJ mol^{-1} nm^{-2})**
Zn_5	O_3	2.01487	122.16486	0.201487	102227.5548
Zn_5	O_4	2.01487	122.15424	0.201487	102218.6680
H_2	C_1	1.08142	357.44038	0.108142	299106.1100
C_1	O_3	1.26901	714.55744	0.126901	597941.6658
C_1	C_1	1.45800	391.66951	0.145800	327749.0460
C_1	C_1	1.37926	462.65505	0.137926	387149.7458

Figure S2 shows the bond energy parity plot evaluated across all simulation steps, accounting for every bond type present in the system. Because the bond parameters do not require any empirical fitting—relying strictly on analytical unit and functional formulation conversion—the alignment is exact, yielding a perfect correlation coefficient ($R^2 = 1.0$).

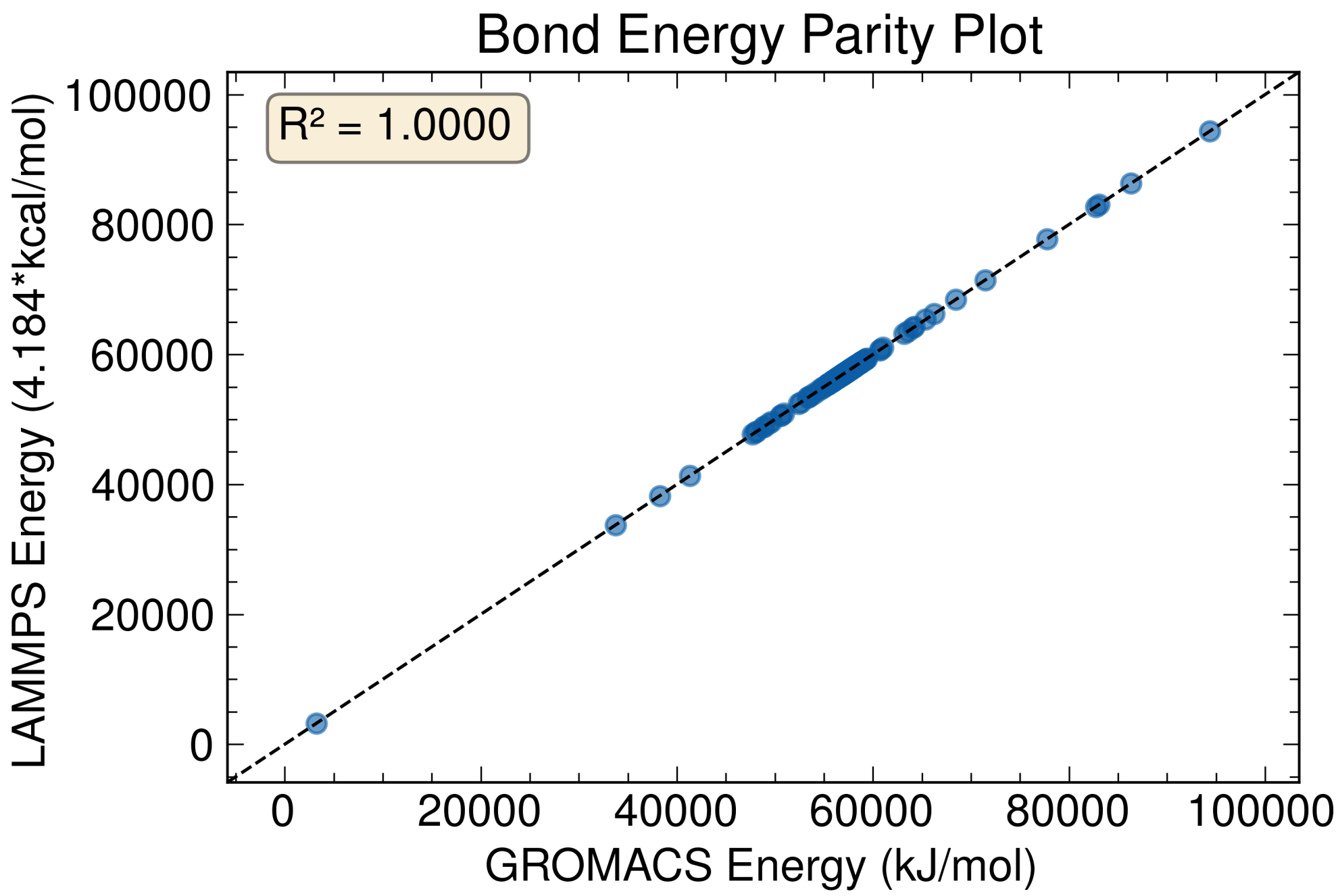

Figure S2: Bond energy parity plot comparing LAMMPS and GROMACS potential energy values across all trajectory frames.

Angles

The LAMMPS `fourier` angle style is defined by the following potential expression:

$$E = K\left[C_0 + C_1\cos(\theta) + C_2\cos(2\theta)\right] \tag{3}$$

where K is the energy prefactor, and C_0, C_1, and C_2 are the dimensionless Fourier expansion coefficients. Conversely, in GROMACS, the corresponding structural angle interactions are represented using the GROMOS-96 functional style (function type 2):

$$V_a(\theta_{ijk}) = \frac{1}{2}k^{\theta}_{ijk}\left[\cos(\theta_{ijk}) - \cos(\theta^0_{ijk})\right]^2 \tag{4}$$

where k^{θ}_{ijk} represents the force constant and θ^0_{ijk} is the equilibrium reference angle.

To initialize the numerical optimization, the initial guess values were set using the heuristics:

$$k_{\text{GROMACS, init}} = \frac{K_{\text{LAMMPS}}}{1000} \tag{5}$$

and

$$\theta^0_{\text{GROMACS, init}} = \theta_{\text{LAMMPS, min}} \times \frac{\pi}{180} \tag{6}$$

where $\theta_{\text{LAMMPS, min}}$ represents the first minimum energy angle obtained from the LAMMPS potential surface. Figure S3 illustrates the potential energy alignment achieved using these optimized parameters for the specific O_3-Zn_5-O_4 angle interaction.

The remaining mapped and converted parameters are summarized in Table S2.

Figure S4 shows the angle energy parity plot evaluated across all simulation steps, accounting for every unique angle type present in the system. Because the angle parameters were derived using a numerical optimization mapping protocol rather than a direct linear conversion, small residual variances may exist; however, the alignment shows exceptional agreement, approaching an ideal correlation.

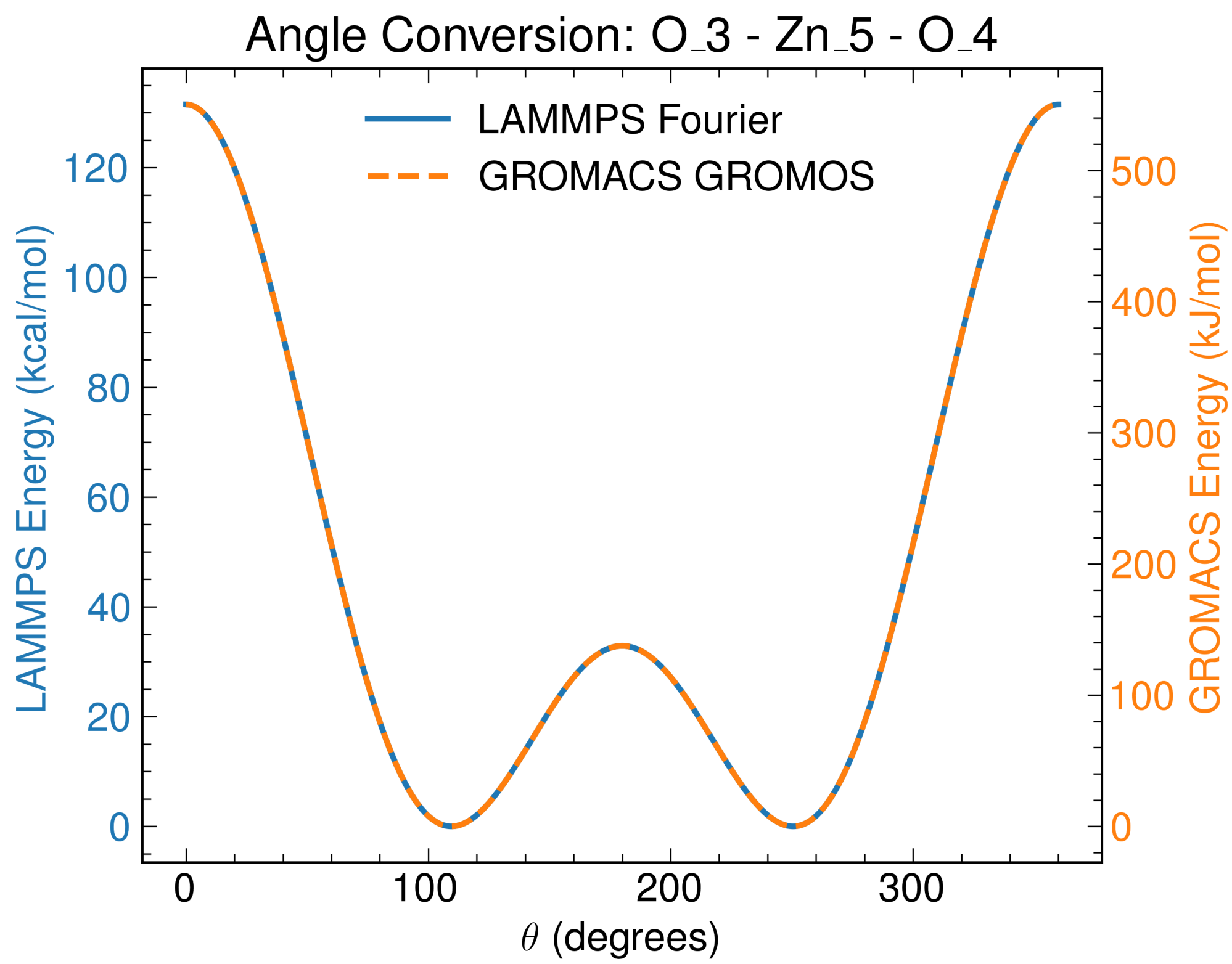

Figure S3: Potential energy profile alignment between LAMMPS Fourier and GROMACS GROMOS-96 angle formulations for the O_3-Zn_5-O_4 interaction after numerical optimization.

Table S2: Mapped equilibrium angles (θ_0), force constants (K, k^{θ}), and Fourier coefficients (C_n) optimized between LAMMPS and GROMACS configurations.

			LAMMPS (Fourier)				GROMACS (GROMOS-96)	
**Atom** i	**Atom** j	**Atom** k	K **(kcal mol^{-1})**	C_0	C_1	C_2	θ_0 **(deg)**	k^{θ} **(kJ mol^{-1})**
O_3	Zn_5	O_4	131.49574	0.34375	0.37500	0.28125	109.4710	618.9505
O_3	Zn_5	O_3	131.50718	0.34375	0.37500	0.28125	109.4710	619.0043
O_3	C_1	O_3	413.55685	0.50000	0.66667	0.33333	10.0005	307.0785
C_1	C_1	O_3	275.42849	0.50000	0.66667	0.33333	10.0005	1536.5084
C_1	C_1	C_1	204.36656	0.50000	0.66667	0.33333	10.0005	1140.0816
C_1	C_1	C_1	222.59502	0.50000	0.66667	0.33333	10.0005	141.7711
C_1	C_1	H_	114.57803	0.50000	0.66667	0.33333	39.9995	639.1862
Zn_5	O_4	Zn_5	42.55738	0.34375	0.37500	0.28125	109.4710	100.3176
C_1	O_3	Zn_5	84.43814	0.50000	0.66667	0.33333	39.9995	471.0475

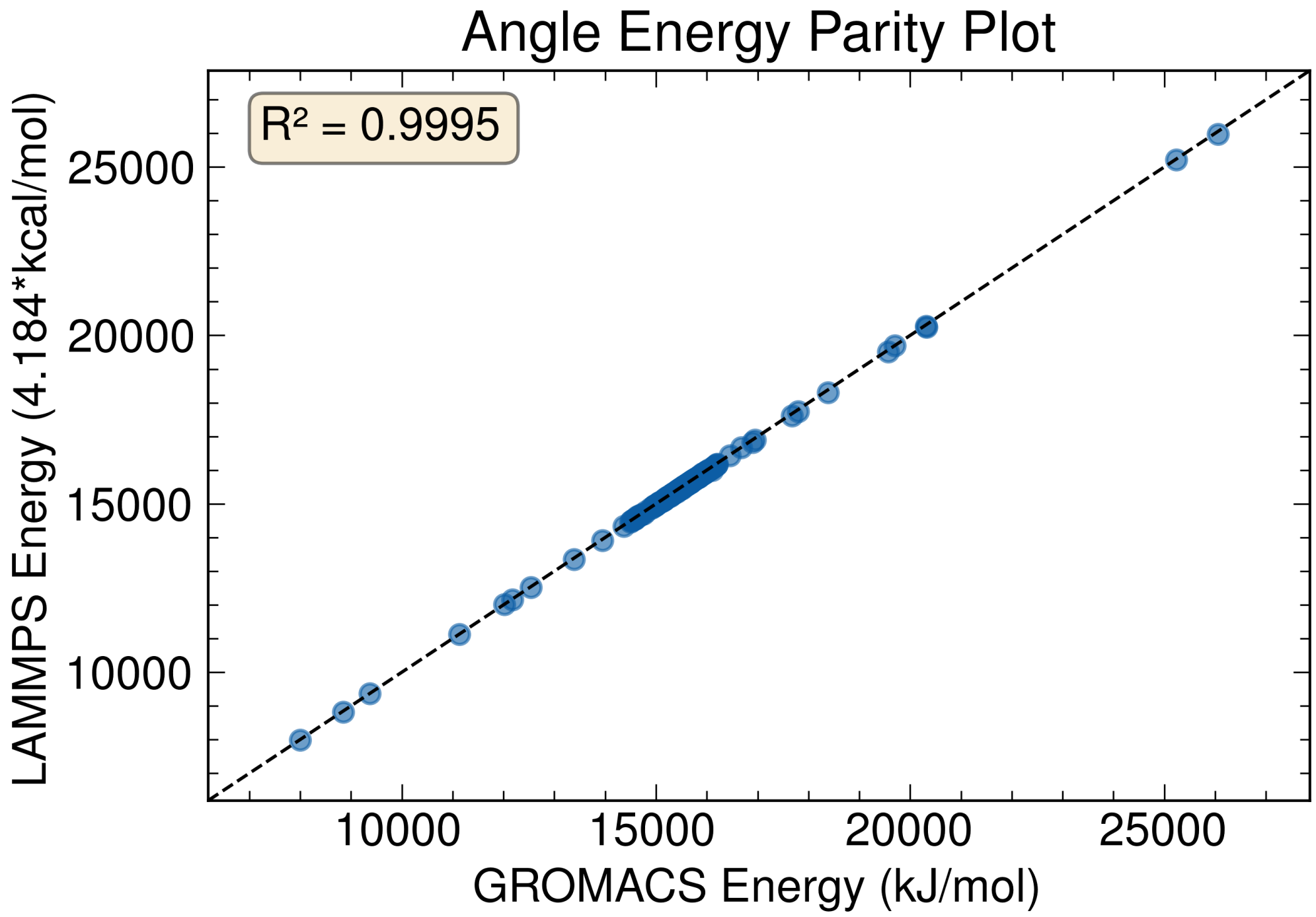

Figure S4: Angle energy parity plot comparing calculated LAMMPS Fourier energies against the optimized GROMACS GROMOS-96 configurations across all trajectory frames.

Dihedrals

The LAMMPS `harmonic` dihedral style uses the potential:

$$E = K\left[1 + d\cos(n\phi)\right] \tag{7}$$

where K is the energy prefactor, d is the sign factor (±1), and n is the multiplicity integer. In GROMACS, similar formulations are handled using the periodic proper dihedral function type 9:

$$V_d(\phi_{ijkl}) = k_\phi\left[1 + \cos(n\phi - \phi_s)\right] \tag{8}$$

where k_ϕ represents the force constant, n is the multiplicity, and ϕ_s is the phase shift angle.

Because these equations share similar formulations, their coefficients can be converted analytically without numerical fitting. The parameters are mapped by scaling the energy from kcal mol^{-1} to kJ mol^{-1}, while the sign factor (d) in LAMMPS is absorbed into the GROMACS phase shift parameter (ϕ_s). If $d = 1$, the phase shift ϕ_s evaluates to 0°; if $d = -1$, it corresponds to a phase shift of 180°, while the structural periodicity value (n) remains unchanged.

Figure S5 illustrates the potential energy alignment using the converted parameters for the O_3 - C_1 - O_3 - Zn_5 dihedral angle.

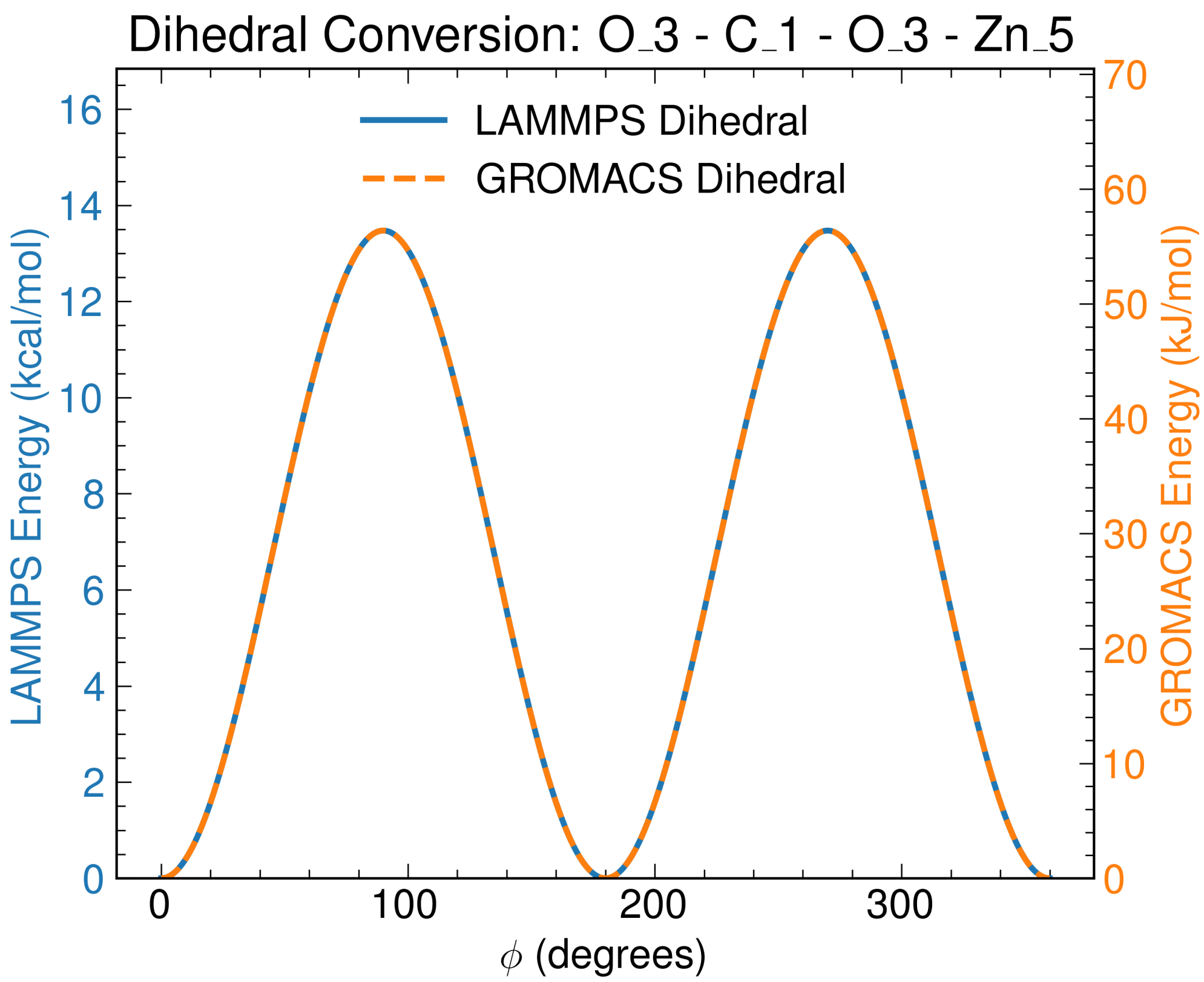

Figure S5: Potential energy profile alignment between LAMMPS and GROMACS periodic dihedral formulations (function type 9) after analytical coefficient translation for the O_3 - C_1 - O_3 - Zn_5 configuration.

The remaining mapped and converted parameters are summarized in Table S3.

Table S3: Mapped phase shifts (ϕ_s), multiplicities (n), and force constants (K, k_ϕ) analytically converted between LAMMPS and GROMACS proper periodic dihedral configurations.

				LAMMPS (Harmonic)			GROMACS (Type 9)		
**Atom i**	**Atom j**	**Atom k**	**Atom l**	K **(kcal mol^{-1})**	d	n	ϕ_s **(deg)**	k_ϕ **(kJ mol^{-1})**	n
O_3	C_1	O_3	Zn_5	6.73711	-1	2	180.0	28.1881	2
C_1	C_1	O_3	Zn_5	6.73711	-1	2	180.0	28.1881	2
O_3	C_1	C_1	C_1	1.25000	-1	2	180.0	5.2300	2
C_1	C_1	C_1	H_2	3.36856	-1	2	180.0	14.0941	2
C_1	C_1	C_1	C_1	3.36856	-1	2	180.0	14.0941	2
H_2	C_1	C_1	H_2	3.36856	-1	2	180.0	14.0941	2
H_2	C_1	C_1	C_1	3.36856	-1	2	180.0	14.0941	2

Figure S6 shows the dihedral energy parity plot evaluated across all simulation steps,

accounting for every unique proper dihedral type present in the system. Because the periodic parameters share matching algebraic formulations and do not require any numerical optimization grid-fitting, the potential curves overlap precisely, yielding a perfect correlation coefficient ($R^2 = 1.0$).

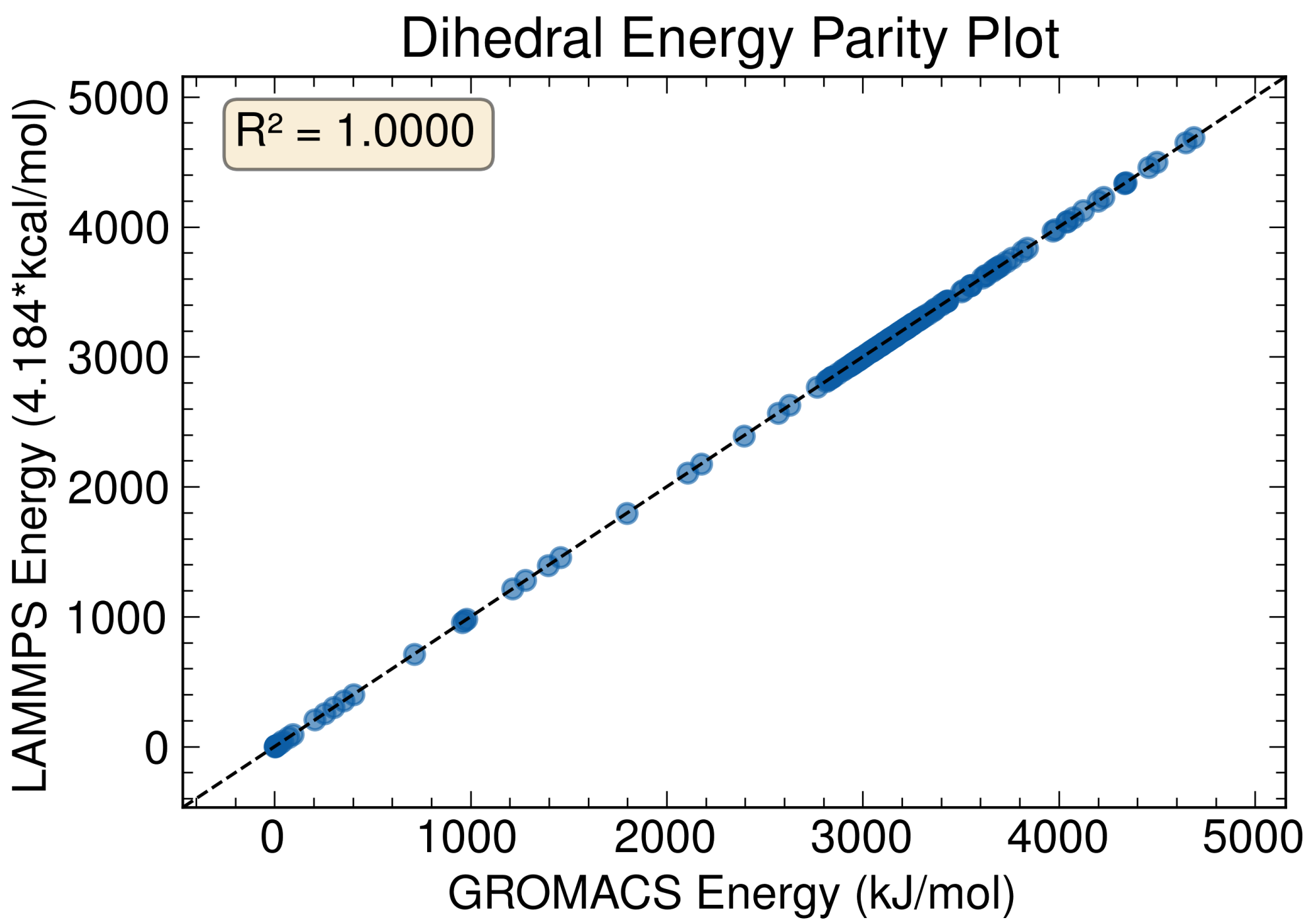

Figure S6: Dihedral energy parity plot comparing calculated LAMMPS harmonic style energies against the analytically translated GROMACS proper periodic function type 9 configurations across all trajectory frames.

Improper Dihedrals

The LAMMPS `fourier` improper style utilizes the following potential:

$$E = K\left[C_0 + C_1\cos(\omega) + C_2\cos(2\omega)\right] \tag{9}$$

where K is the energy prefactor, ω is the improper dihedral angle, and C_0, C_1, and C_2 are the Fourier expansion coefficients. In GROMACS, these out-of-plane configurations are represented using the harmonic improper function type 2:

$$V_{id}(\xi_{ijkl}) = \frac{1}{2}k_\xi(\xi_{ijkl} - \xi_0)^2 \tag{10}$$

where k_ξ is the harmonic force constant and ξ_0 is the equilibrium reference angle.

Because these two mathematical formulations represent entirely different functional spaces, a direct analytical parameter transformation is not possible. Although mapping the LAMMPS expressions into GROMACS periodic proper dihedrals (function type 9) would yield an exact translation, we instead opted to preserve the distinct topological segregation of improper terms by fitting them natively into the GROMACS harmonic framework.

Figure S7 illustrates this localized fitting approach by comparing the energy profiles for a representative C_1-O_3-O_3-C_1 interaction configuration.

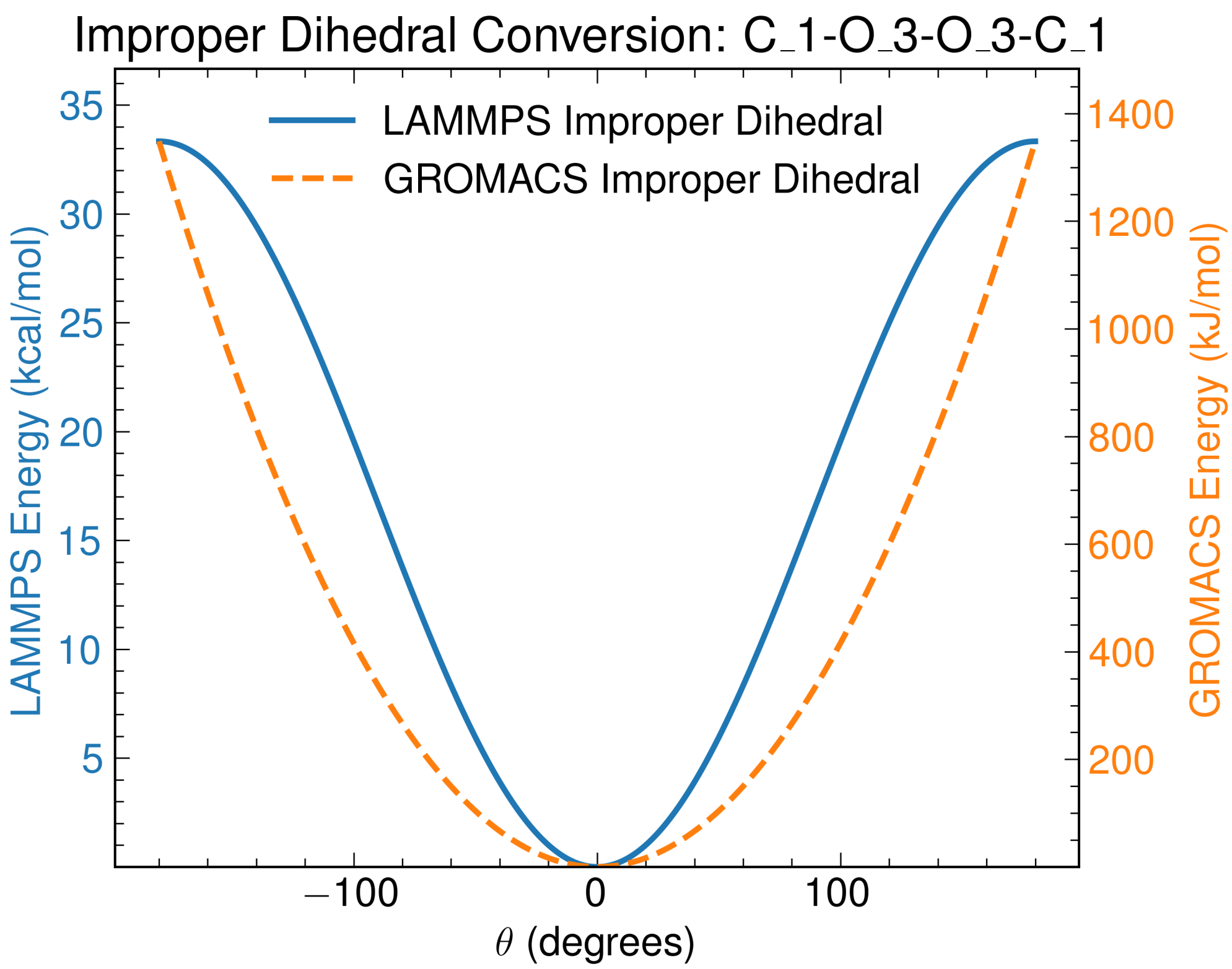

Figure S7: Potential energy profile alignment between the LAMMPS Fourier improper style and the optimized GROMACS harmonic formulation (function type 2) for the C_1-O_3-O_3-C_1 out-of-plane interaction.

To achieve this, a localized numerical optimization procedure was implemented using a least-squares minimization over a physically relevant out-of-plane angular domain ($-\pi/3 \leq \omega \leq \pi/3$ radians). This bounded routine directly minimizes the residual sum of squares between the target harmonic form and the baseline LAMMPS Fourier potential curve (pre-scaled by 4.184 kcal $\rightarrow$ kJ). The resulting parameter pairs mapped via this windowed optimization scheme are compiled in Table S4.

While this harmonic localization introduces a minor geometric approximation for individual out-of-plane potentials at extreme deflections, its isolated impact on the standalone improper potential energies across trajectory configurations is captured in Figure S8.

Table S4: Mapped equilibrium angles (ξ_0), expansion coefficients (C_n), and optimized harmonic force constants (K, k_ξ) converted between LAMMPS and GROMACS improper topologies.

				LAMMPS (Fourier)				GROMACS (Type 2)	
**Atom i**	**Atom j**	**Atom k**	**Atom l**	K **(kcal mol^{-1})**	C_0	C_1	C_2	ξ_0 **(deg)**	k_ξ **(kJ mol^{-1} rad^{-2})**
C_1	O_3	O_3	C_1	16.66667	1.00000	-1.00000	0.00000	0.0	273.2511
C_1	C_1	O_3	O_3	16.66667	1.00000	-1.00000	0.00000	0.0	273.2511
C_1	O_3	C_1	O_3	16.66667	1.00000	-1.00000	0.00000	0.0	273.2511
C_1	C_1	C_1	C_1	2.00000	1.00000	-1.00000	0.00000	0.0	32.7901
C_1	H_2	C_1	C_1	2.00000	1.00000	-1.00000	0.00000	0.0	32.7901
C_1	C_1	H_2	C_1	2.00000	1.00000	-1.00000	0.00000	0.0	32.7901

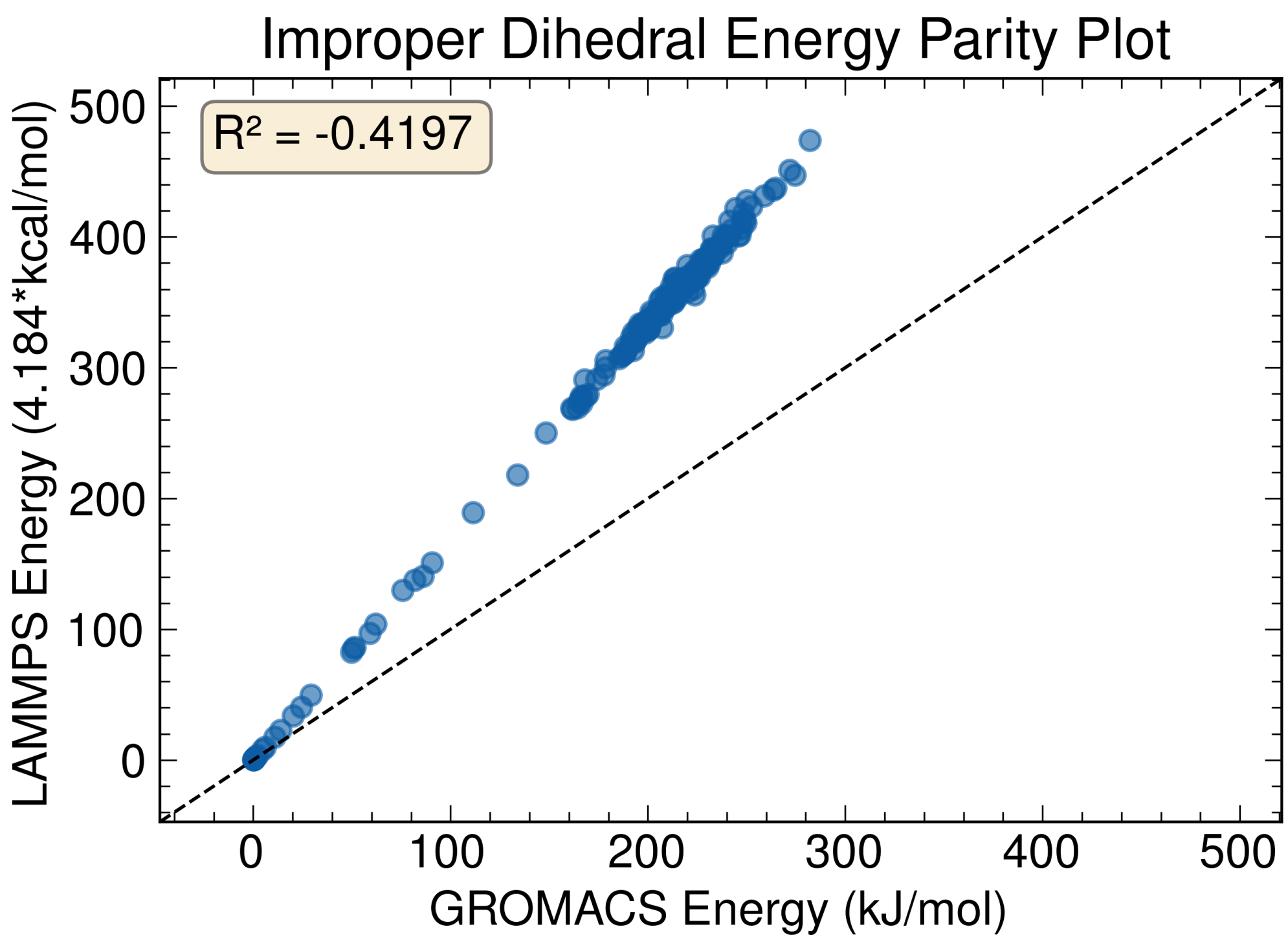

Figure S8: Improper dihedral energy parity plot mapping isolated LAMMPS Fourier out-of-plane potential contributions against the fitted GROMACS harmonic counterparts.

Crucially, the cumulative impact of this approximation on the overall macromolecular system configuration remains negligible. To verify the cumulative structural reliability, Figure S9 presents a comprehensive parity plot tracking the total summation of all bonded energy contributions across every simulated trajectory step. Despite the localized approximation utilized for the improper terms, the global ensemble agreement tracks with exceptional accuracy, showing a near-perfect correlation coefficient ($R^2 = 0.9998$).

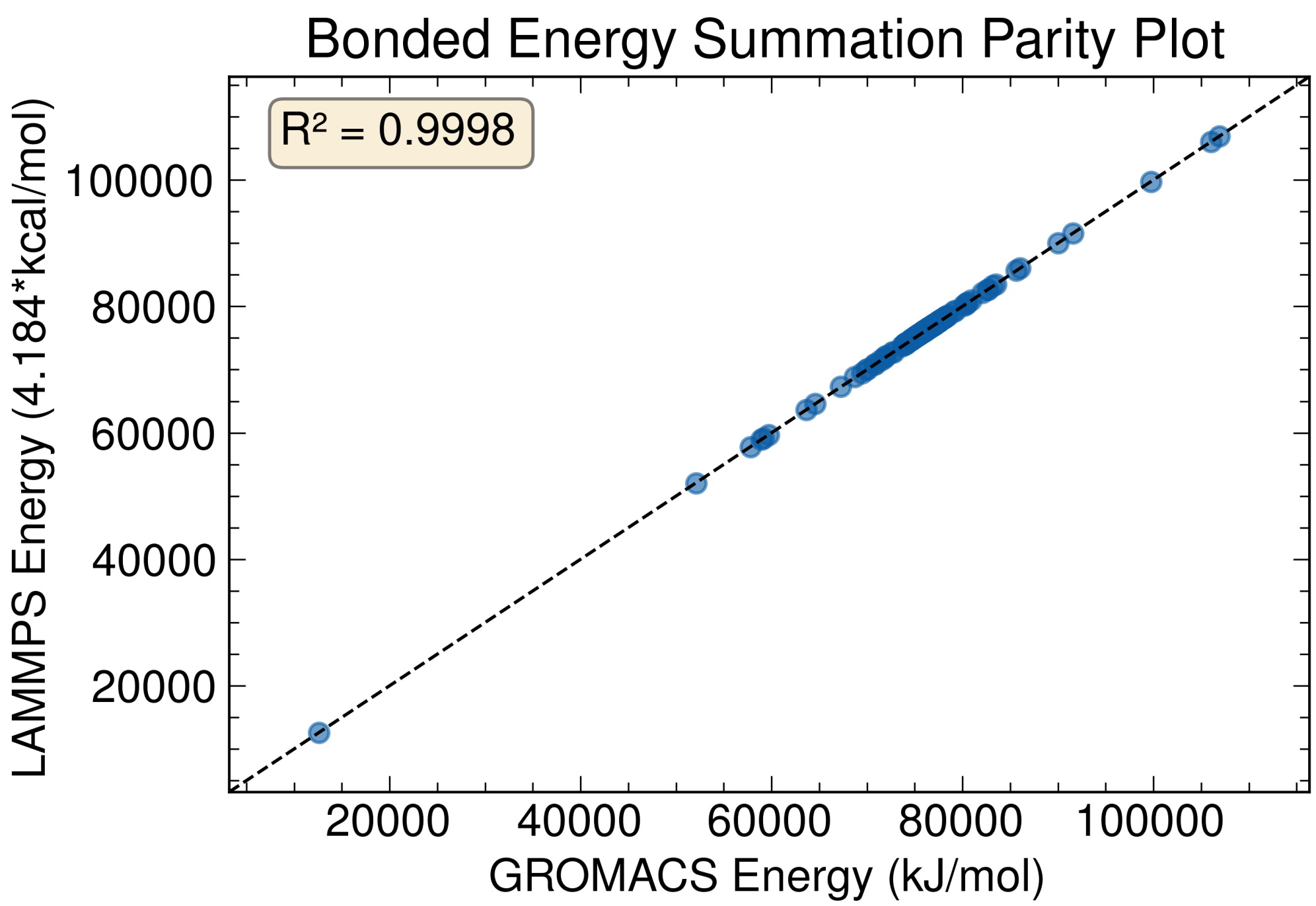

Figure S9: Global bonded energy parity plot highlighting the cumulative summation agreement across all concurrent bond, angle, dihedral, and optimized improper interactions between LAMMPS and GROMACS trajectory profiles.

Case 2

Bonds and Angles

Within the IFF-CVFF force field framework, both bond stretching and angle bending interactions are governed by standard harmonic functional forms. Consequently, the governing potential equations and parameter conversions follow the identical analytical scaling procedures detailed for the harmonic bond style in Case 1.

Because both simulation engines utilize identical algebraic formulations, the mapping requires only an analytical unit conversion. The explicitly mapped parameter sets for the evaluated topologies are consolidated in Table S5.

To verify the rigorous mathematical equivalence of these implementations within our conversion engine, Figure S10 illustrates the energy parity plots for both the bond and angle potential components across all recorded simulation steps. Owing to the shared functional

Table S5: Analytical parameter conversion for harmonic bonds (b_0, k_b) and angles (θ_0, k_θ) between LAMMPS and GROMACS configurations using the IFF-CVFF force field.

			LAMMPS (Harmonic)		GROMACS (Function Type 1)
**Interaction**	**Atoms**	**Ref. Value**	**Force Const.**	**Ref. Value**	**Force Const.**
**Bond**		r_0 (Å)	K (kcal mol^{-1} Å^{-2})	b_0 (nm)	k_b (kJ mol^{-1} nm^{-2})
	oc23 – sc4	1.6800	285.0000	0.168000	238488.00
**Angle**		θ_0 (deg)	K (kcal mol^{-1} rad^{-2})	θ_0 (deg)	k_θ (kJ mol^{-1} rad^{-2})
	sc4 – oc23 – sc4	149.00	100.0000	149.00	836.80
	oc23 – sc4 – oc23	109.50	100.0000	109.50	836.80

paradigms between the engines, the analytical conversion yields an exact mapping, resulting in an ideal overlap with a correlation coefficient of $R^2 = 1.0$.

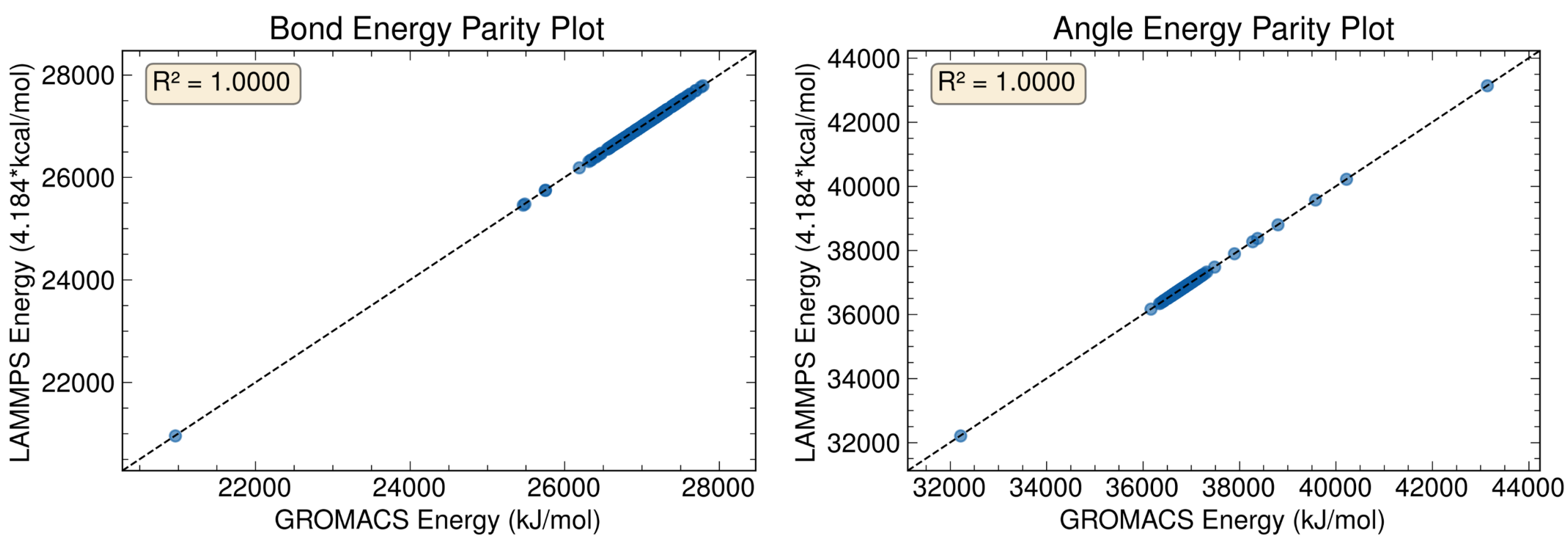

Figure S10: Potential energy parity evaluation under the IFF-CVFF framework (Case 2) across all trajectory steps for (a) harmonic bond stretching and (b) harmonic angle bending components.

Dihedrals and Impropers

The proper dihedral interactions within the IFF-CVFF framework utilize the periodic proper dihedral functional form, identical to the formalism presented for Case 1. However, for the specific solid-phase configurations evaluated in this case study, the only parameterized dihedral is sc4–oc23–sc4–oc23.

This interaction carries an assigned force constant prefactor of $K = 0.0$ kcal mol^{-1}. Consequently, this zero-magnitude assignment produces no net potential energy contributions during structural evaluations, rendering the generation of corresponding energy parity plots for this component irrelevant.

The improper interactions within the IFF-CVFF framework utilize the LAMMPS `cvff` improper style, which shares an identical mathematical functional form with the classic harmonic proper dihedral style. Because of this structural alignment between the potentials, these terms do not require the localized numerical grid-fitting optimization used for Case 1 impropers. Instead, the parameters were mapped directly using the exact analytical translation protocol derived for the Case 1 proper dihedrals section.

Under this scheme, the energy terms are converted via analytical scaling from kcal mol^{-1} to kJ mol^{-1}, with the structural periodicity and sign factors preserved directly across the matching functional spaces.

References

(S1) Rappé, A. K.; Casewit, C. J.; Colwell, K. S.; Goddard, W. A.; Skiff, W. M. UFF, a full periodic table force field for molecular mechanics and molecular dynamics simulations. *Journal of the American Chemical Society* **1992**, *114*, 10024–10035.

(S2) Heinz, H.; Lin, T.-J.; Kishore Mishra, R.; Emami, F. S. Thermodynamically Consistent Force Fields for the Assembly of Inorganic, Organic, and Biological Nanostructures: The INTERFACE Force Field. *Langmuir* **2013**, *29*, 1754–1765.

(S3) LAMMPS Documentation (30 Mar 2026 version); LAMMPS documentation — docs.lammps.org. `https://docs.lammps.org/{M}anual.html`, [Accessed 17-06-2026].

(S4) Reference Manual - GROMACS 2026.2 documentation — manual.gromacs.org. `https://manual.gromacs.org/2026.2/reference-manual/index.html`, [Accessed 17-06-2026].

(S5) Thompson, A. P.; Aktulga, H. M.; Berger, R.; Bolintineanu, D. S.; Brown, W. M.; Crozier, P. S.; in 't Veld, P. J.; Kohlmeyer, A.; Moore, S. G.; Nguyen, T. D.; Shan, R. et al. LAMMPS - a flexible simulation tool for particle-based materials modeling at the atomic, meso, and continuum scales. *Computer Physics Communications* **2022**, *271*, 108171.

(S6) Humphrey, W.; Dalke, A.; Schulten, K. VMD – Visual Molecular Dynamics. *Journal of Molecular Graphics* **1996**, *14*, 33–38.

(S7) Abraham, M. J.; Murtola, T.; Schulz, R.; Páll, S.; Smith, J. C.; Hess, B.; Lindahl, E.

GROMACS: High performance molecular simulations through multi-level parallelism from laptops to supercomputers. *SoftwareX* **2015**, *1-2*, 19–25.
